# Supplementary material for: Metabolic constraints on the evolution of antibiotic resistance
Source: Mol Syst Biol. 2017 Mar 6;13(3):917. doi: 10.15252/msb.20167028 (PMC5371735; doi:10.15252/msb.20167028)
Supplement: Supplementary file 1 — Appendix [file MSB-13-917-s001.pdf]

Appendix

Metabolic constraints on the evolution of antibiotic resistance.

Authors: Mattia Zampieri, Tim Enke, Victor Chubukov, Vito Ricci, Laura Piddock and Uwe Sauer

Table of contents:

Appendix Figures 2

Appendix References 19

Tables available in separate files:

Dataset EV1 Estimation of mutation rate

Dataset EV2 Metabolome profiling dataset

Dataset EV3 R^2 estimated from sigmoidal fitting analysis

Dataset EV4 EMC analysis

Dataset EV5 Measured and predicted fluxes in glucose evolved *E. coli*

Dataset EV6 Whole genome sequencing

Dataset EV7 Difference in oxygen uptake rates in strains with and without mutations

Supplementary Results

Appendix Figures

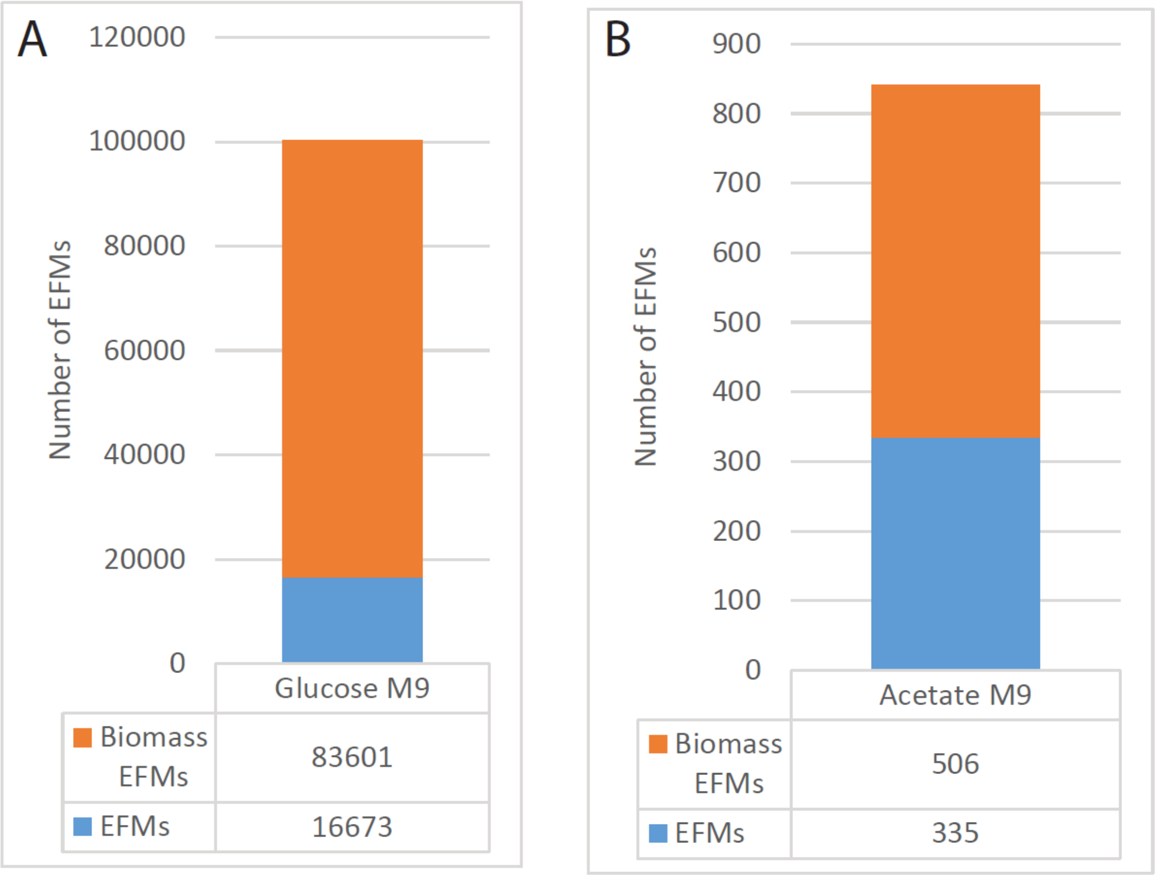

Figure S1: Elementary Flux Modes of *E. coli* central carbon metabolic model. The elementary flux modes individuated in a stoichiometric model of central carbon metabolism in *E. coli* when growing in a glucose minimal medium (A) and acetate minimal medium (B). EFMs supporting growth are highlighted in orange.

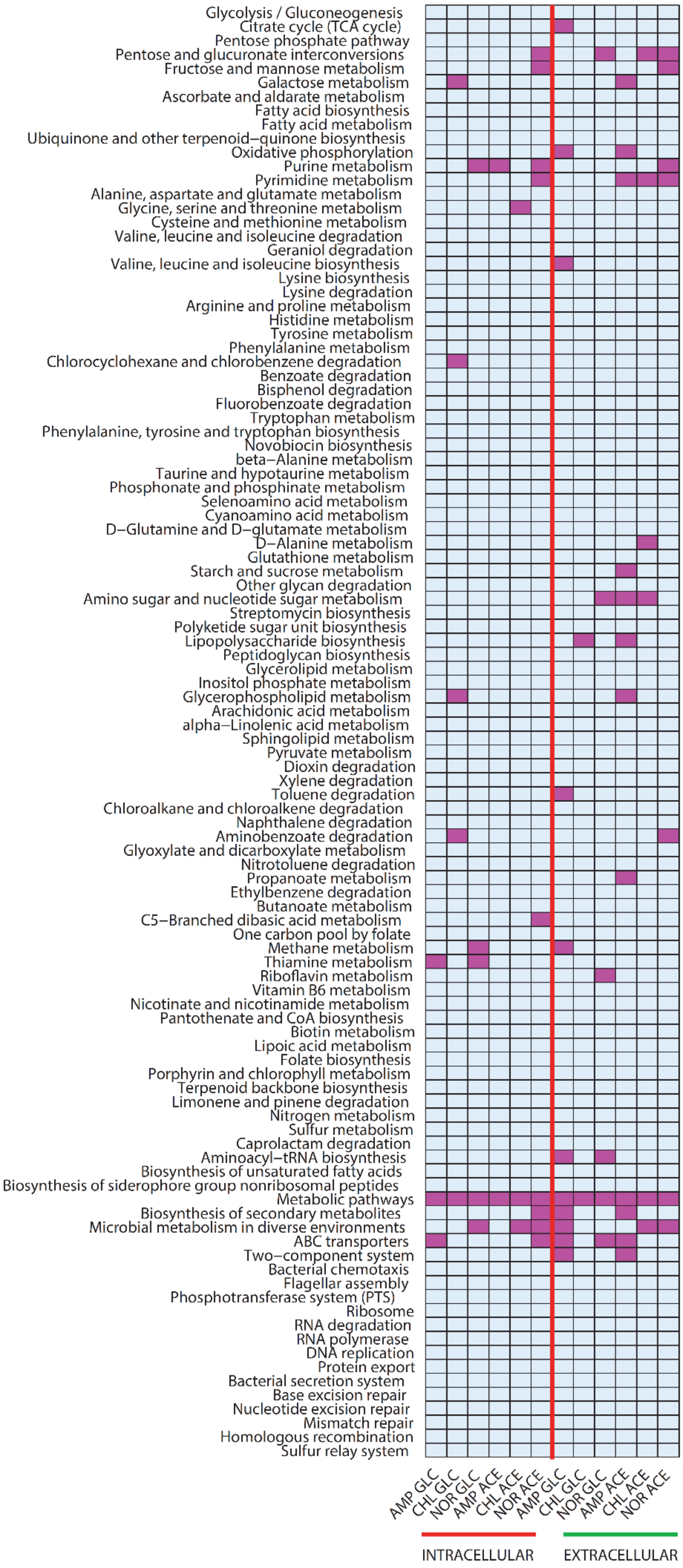

Figure S2: The R^2 values derived from the sigmoidal fitting analysis, were averaged across the 4 lineages evolved under a common selective pressure (medium - antibiotic). KEGG enrichment analysis was performed using the method described in (König *et al*, 2007). Enrichment analysis was performed separately for intracellular and extracellular metabolome profiles. For each condition, annotated metabolites were ranked according to their averaged R^2 values. A statistical score that models the probability of overrepresented metabolite sets in different KEGG pathways is based on the collective activities of multiple metabolites following the approach described in (König *et al*, 2007). Specifically, the rank distribution of all metabolites belonging to the same pathway is examined and a *p*-value is assigned following an iterative hypergeometric test (König *et al*, 2007). The iterative hypergeometric test evaluates the significance of the rank distribution of all metabolites annotated to the same pathway, indicating the statistical relevance of metabolites in a common cellular process being distributed toward the top ranking ones. P-values were corrected for multiple test by means of q-value correction (Storey, 2002). Pathways with a q-value significance of overrepresented altered metabolites lower or equal to 0.01 are highlighted in purple.

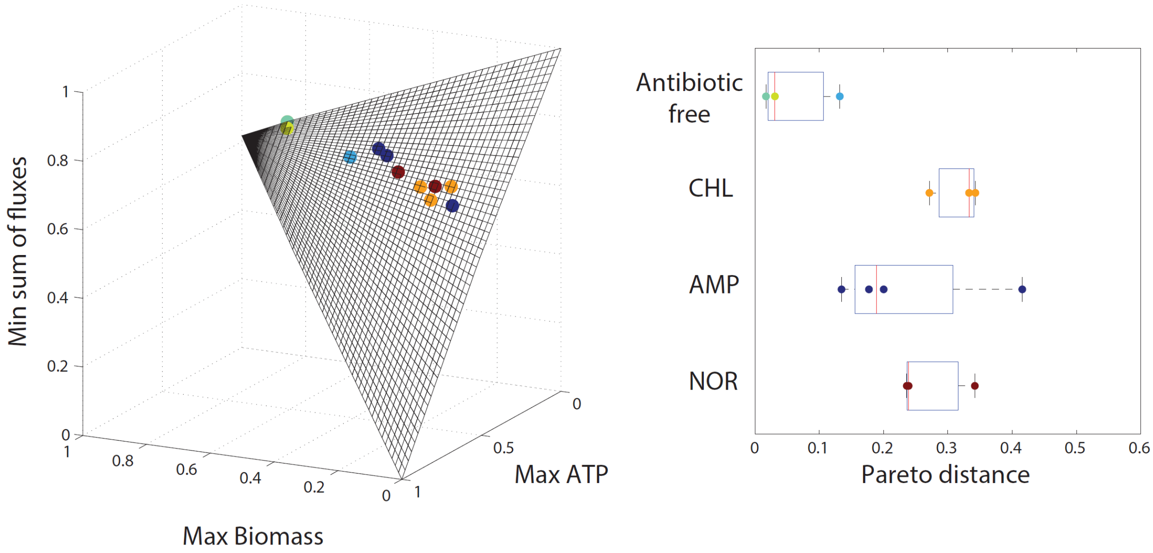

Figure S3: Pareto optimality. (Left-panel) The pareto surface estimated by simulating a glucose minimal medium is shown as a 3D surface. Each color-coded dot corresponds to one of the evolved strains. (Right-panel) minimum distance of the metabolic flux distributions in the evolved strains to the pareto optima is shown for each strain. Evolved strains have been grouped accordingly to the antibiotic selective pressure.

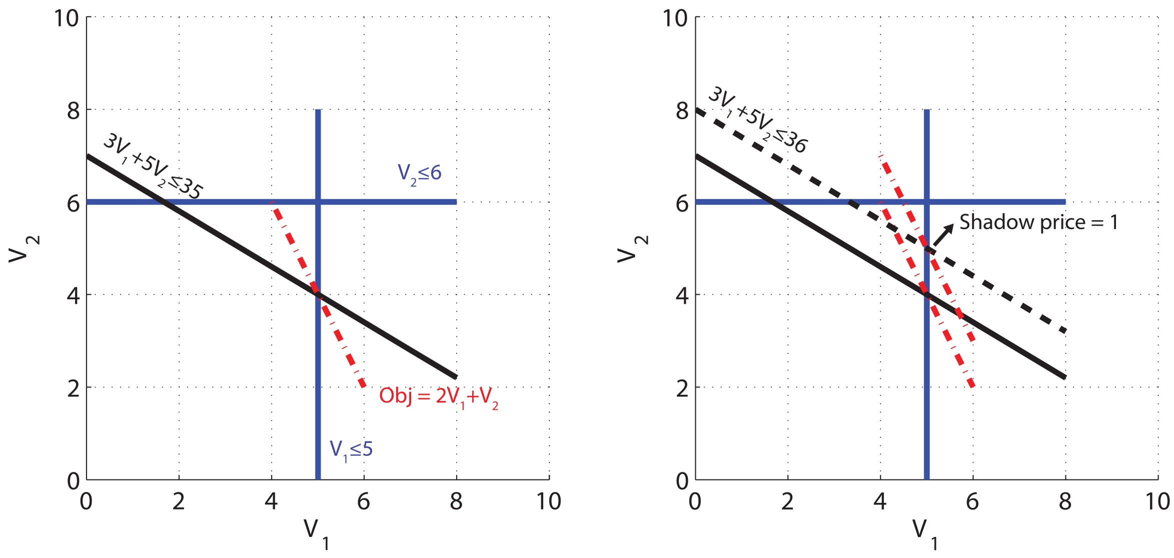

Figure S4: Estimation of shadow prices. In maximization problems, the constraints can often be described as restrictions on the amount of resources available, and the objective as a measure of profit. Here we show a hypothetical problem where a set of linear constraints (thick blue and black lines) limits the availability of two resources (V_1 and V_2). Only one combination of V_1 and V_2 is optimal for maximizing the sum of $2V_1+V_2$ (objective function red dashed line). The shadow price associated with a particular constraint reveals how much the optimal value of the objective would increase per unit increase in the amount of resource available (right panel). In other words, the shadow price associated with a resource tells how much more profit you would get by increasing the amount of that resource by one unit. In this particular problem, small changes on the upper limit of available resource V_2 is irrelevant for the objective.

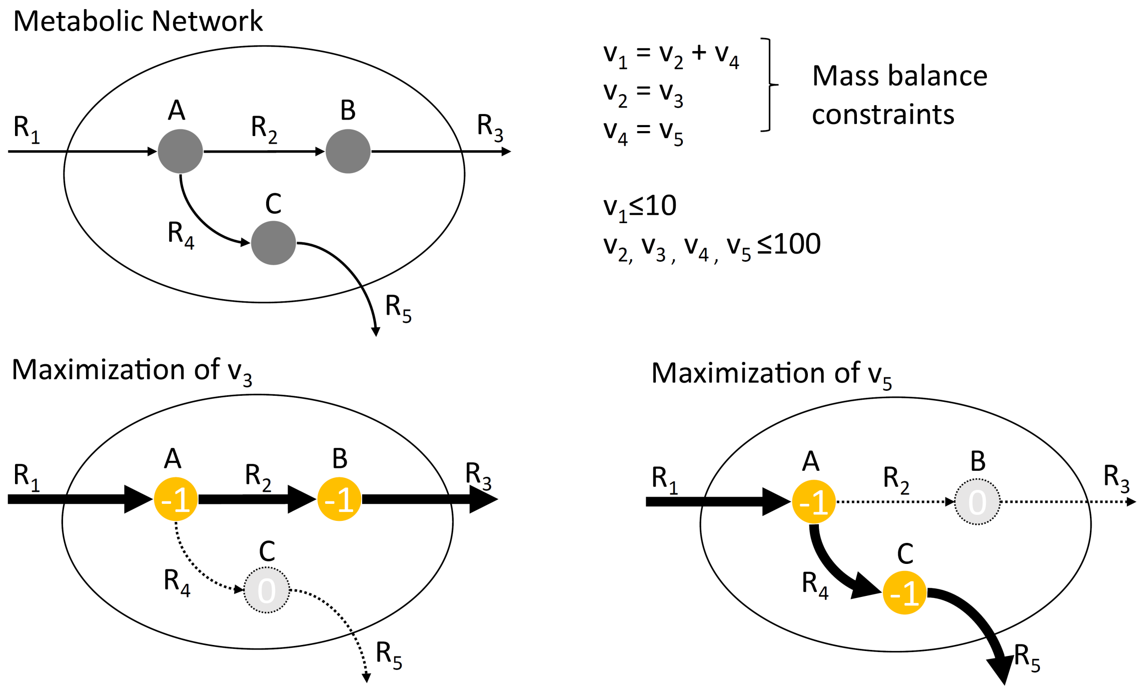

Figure S5: Sensitivity parameters - Shadow prices estimated from the dual solution to the FBA problem. (A) Here we show a toy model of a metabolic network consisting of 3 metabolites and 5 reactions. (B-C) Under assumption of steady-state and R1 to be limiting, we can calculate the shadow prices associated to metabolite A, B and C when maximizing the outputs R5 or R3. A negative shadow price (i.e. -1) reflects the sensitivity of the objective function to an imbalance of the corresponding metabolite (i.e. sink flux). In this example maximization of fluxes through R5 or R3 yields different shadow prices as reported in the figure.

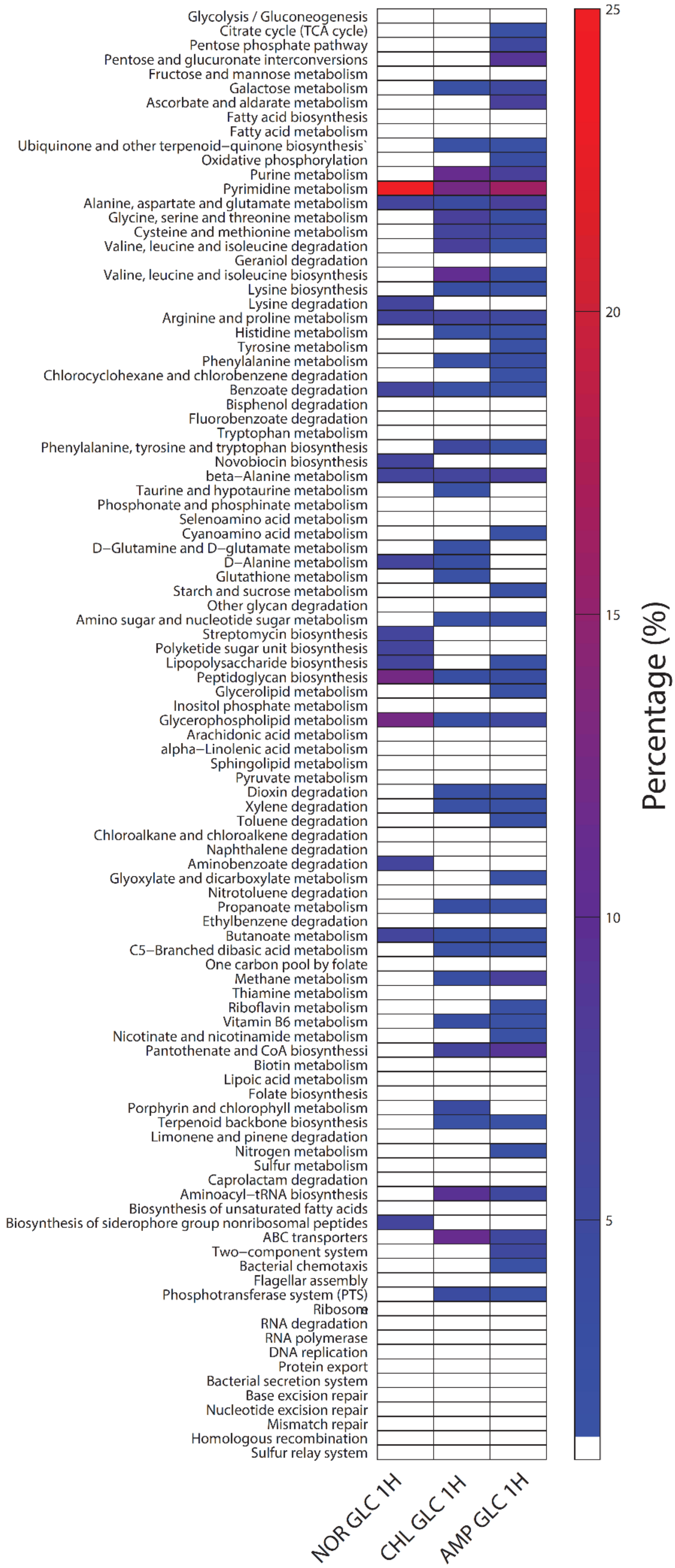

Figure S6: Metabolic changes after one hour from drug exposure. For all three antibiotics, we calculated the number of significantly changed metabolites within each metabolic pathway relative to the total number of significant changes.

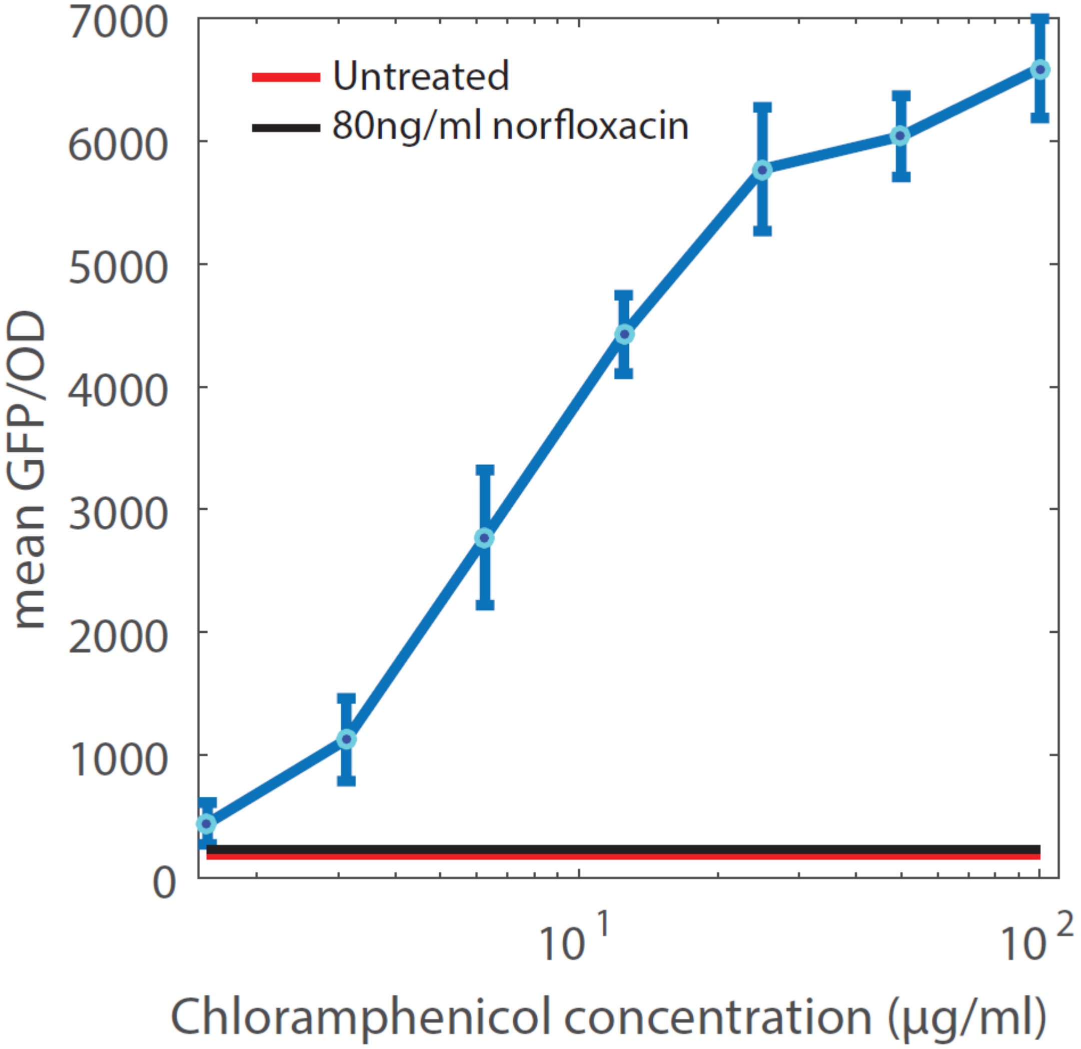

Figure S7: Measurement of *acrB* expression. AcrAB promoter activity was monitored using a GFP reporter (Bumann & Valdivia, 2007). Wildtype *E. coli* was treated with different concentrations of chloramphenicol (blu line) and 80 ng/ml of norfloxacin (black line). The average GFP levels divided by the corresponding OD values during exponential growth are shown. Basal AcrAB promoter activity was measured upon normal growth without antibiotics (red line). We observed that the higher is the chloramphenicol concertation the higher is the induction of the AcrAB expression, up to a plateau around 25 µg/ml. Differently, norfloxacin induces only a nonsignificant increase of the promoter activity.

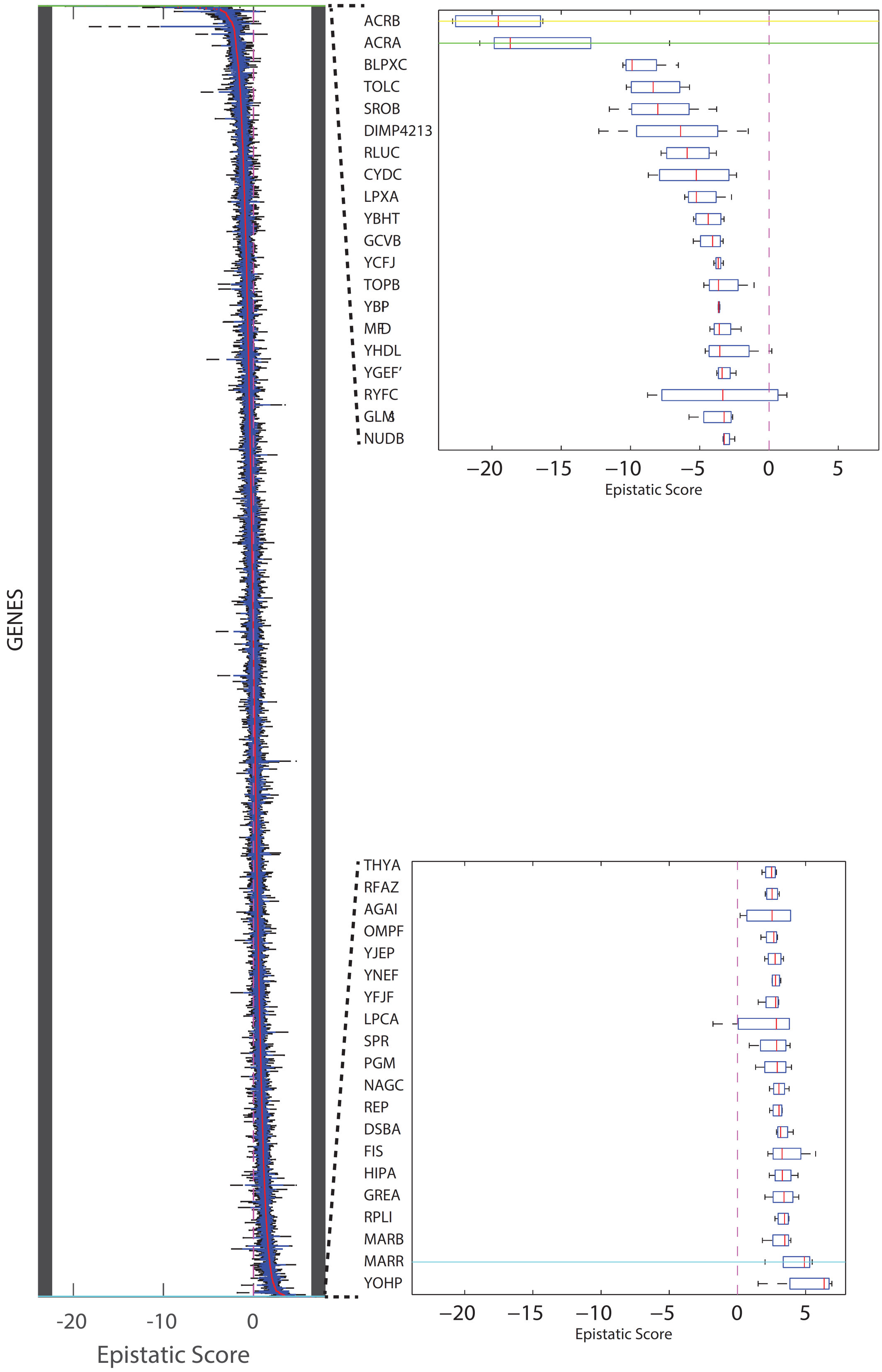

Figure S8: Epistatic interactions scores reported in (Nichols *et al*, 2011) between chloramphenicol and single gene deletion mutants of *E. coli*. Boxplots represent the median and first and third quartiles of the different dosages

tested for chloramphenicol. Top/Bottom 20 genes are reported in the figure insets on the right. Blu, yellow and green line represent respectively *ΔmarR*, *ΔacrB* and *ΔacrA*.

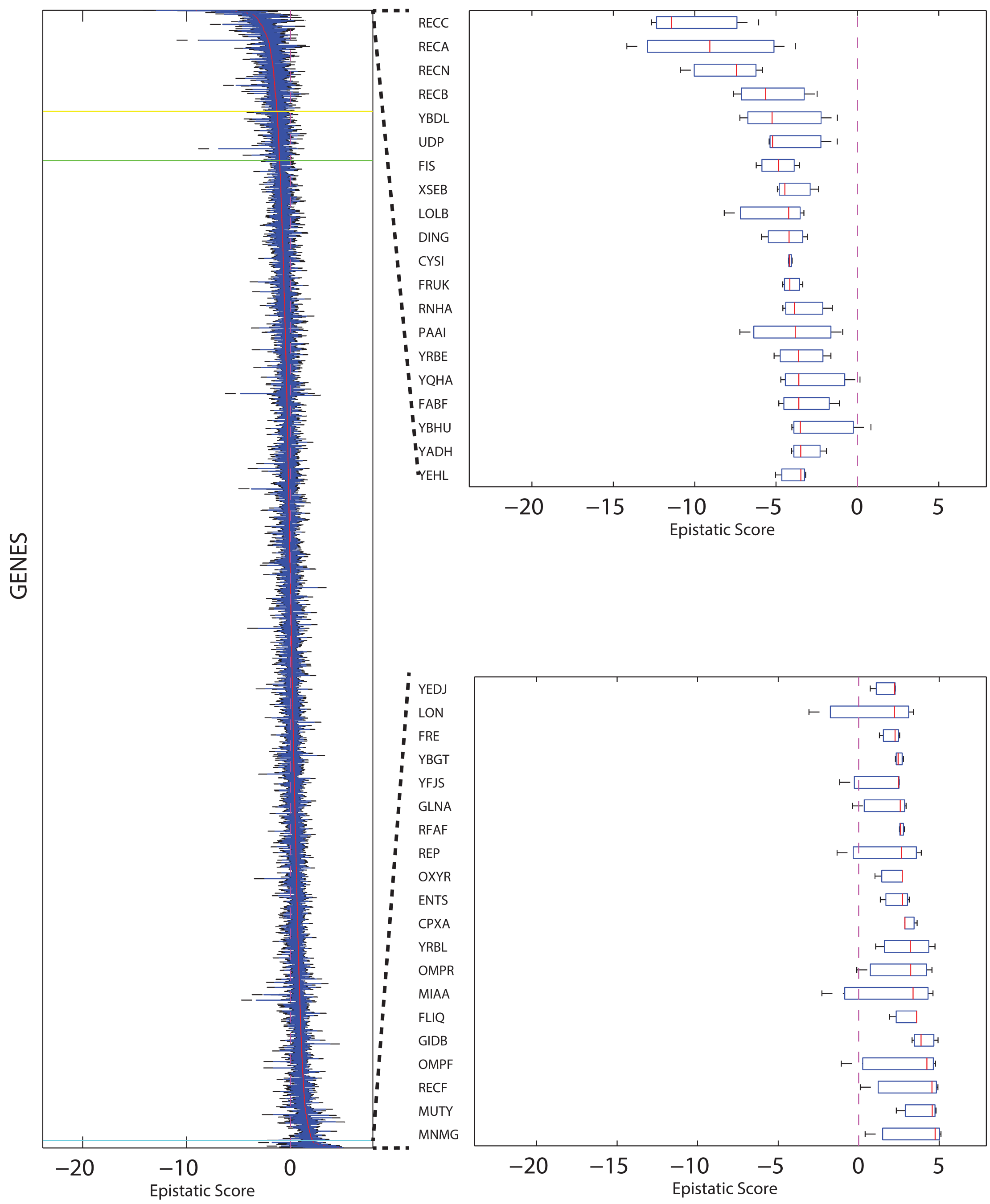

Figure S9: Epistatic interactions scores reported in (Nichols *et al*, 2011) between norfloxacin and single gene deletion mutants of *E. coli*. Boxplots represent the median and first and third quartiles of the different dosages tested for chloramphenicol. Top/Bottom 20 genes are reported in the figure insets on the right. Blu, yellow and green line represent respectively *ΔmarR*,

ΔacrB and *ΔacrA*. Differently from chloramphenicol, other gene-knockout mutants seem to be more effective in mediating tolerance to norfloxacin.

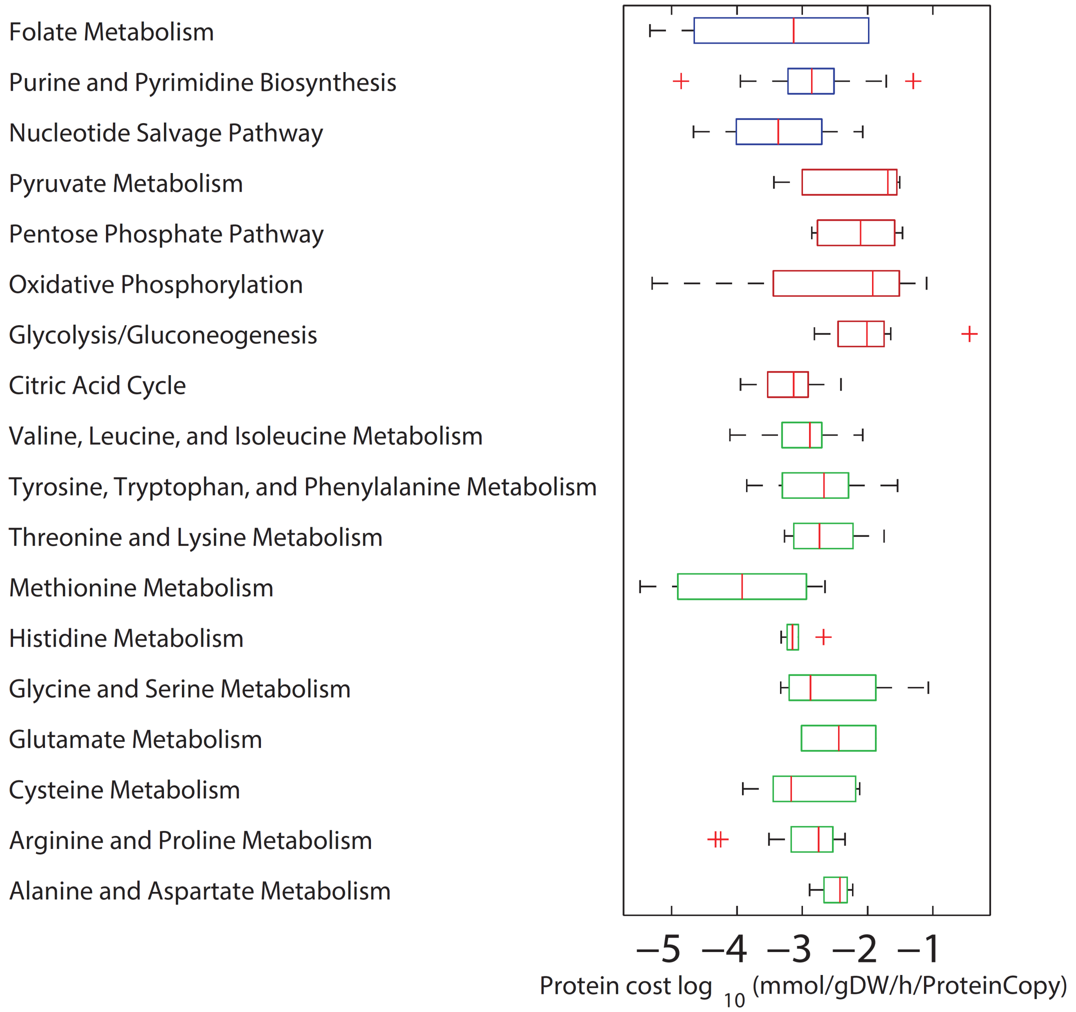

Figure S10: Enzyme cost in central carbon metabolism. Protein copy numbers for all annotated enzyme in the genome-scale model of *E. coli* metabolism (Orth *et al*, 2011) were collected from (Li *et al*, 2014). Estimated fluxes in Fig. S6 in a glucose minimal medium were used to calculate the ratio between flux and corresponding catalyzing enzyme's copy number. This procedure allows to compare how much enzyme is needed to catalyze the same metabolic conversion rate (mmol/gDW/h) across different metabolic reaction. Enzyme belonging to different pathways, according to the metabolic genome scale model (Orth *et al*, 2011) are grouped, and corresponding costs plotted as boxplots, representing the median and first and third quartiles. Nucleotide metabolism related enzymes (blue) are on average the most costly ones. As previously shown in (Li *et al*, 2014), methionine biosynthesis represents by far the amino acids requiring the highest proteome investment (green). TCA cycle

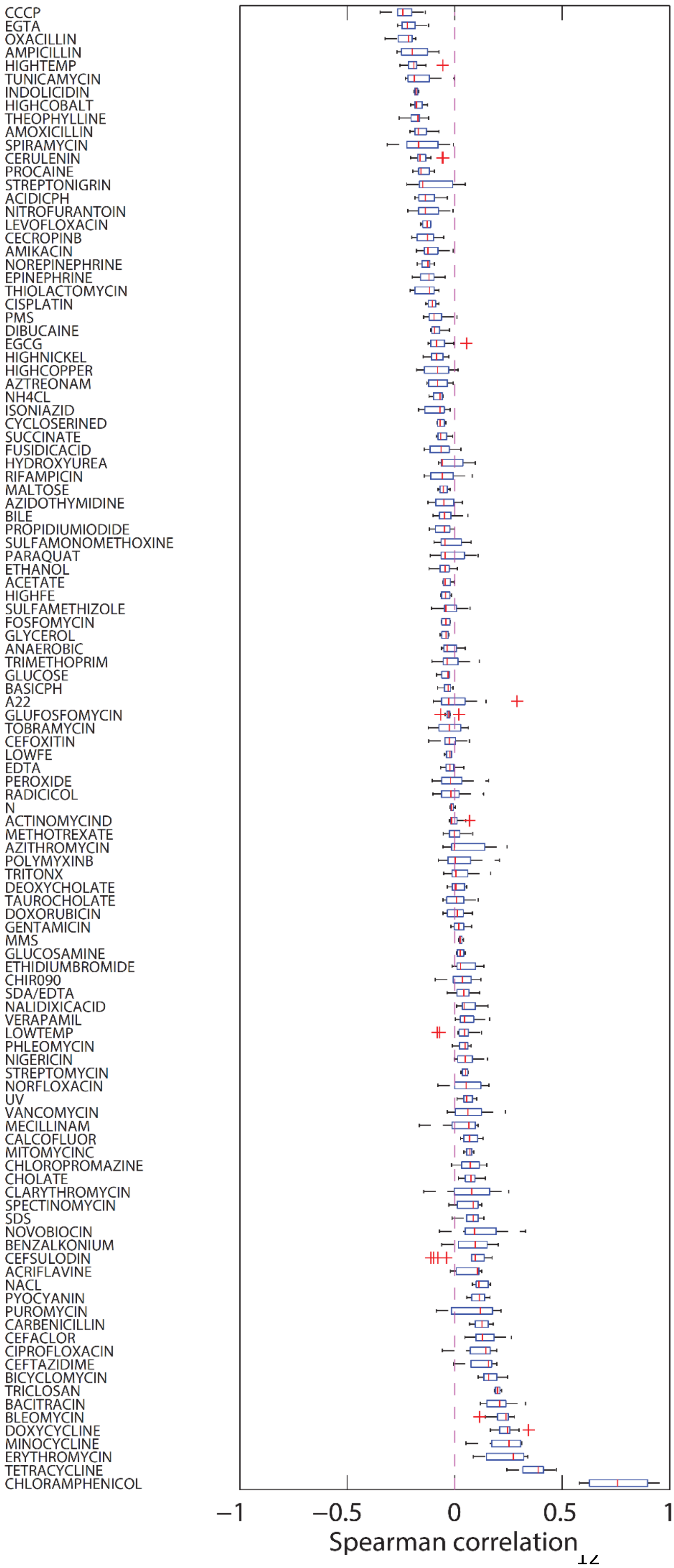

related enzymes are on average almost 5 times more costly then glycolytic enzymes (red boxplots).

Figure S11: Correlated and anti-correlated phenotypic signatures to chloramphenicol. The genetic interaction between ~100 chemical agents (among which chloramphenicol) and ~4000 genes in *E. coli* have been quantitatively characterized in (Nichols *et al*, 2011), using the library of single gene deletions of *E. coli* (Baba *et al*, 2006). Here, pairwise correlation of quantitative fitness measurements between chloramphenicol and the remaining perturbing agents are reported. Since multiple concentrations of the same perturbing agent were tested the box plot reports median and first/third quartiles of Spearman correlation against each perturbing agent/dosages. We observed a tendency for gene knockouts that aggravate the effect of chloramphenicol to buffer the effect of perturbing agents such as EGTA, ampicillin,

amoxicillin or CCCP, while knockouts that buffer the action of chloramphenicol are deleterious for EGTA, ampicillin, amoxicillin or CCCP.

By rearranging the proteome resources to switch from respiratory to more fermentative metabolism, we move from a condition of high proteome cost (due to the expression of costly enzymes in TCA) to low energetic yield (Basan *et al*, 2015).

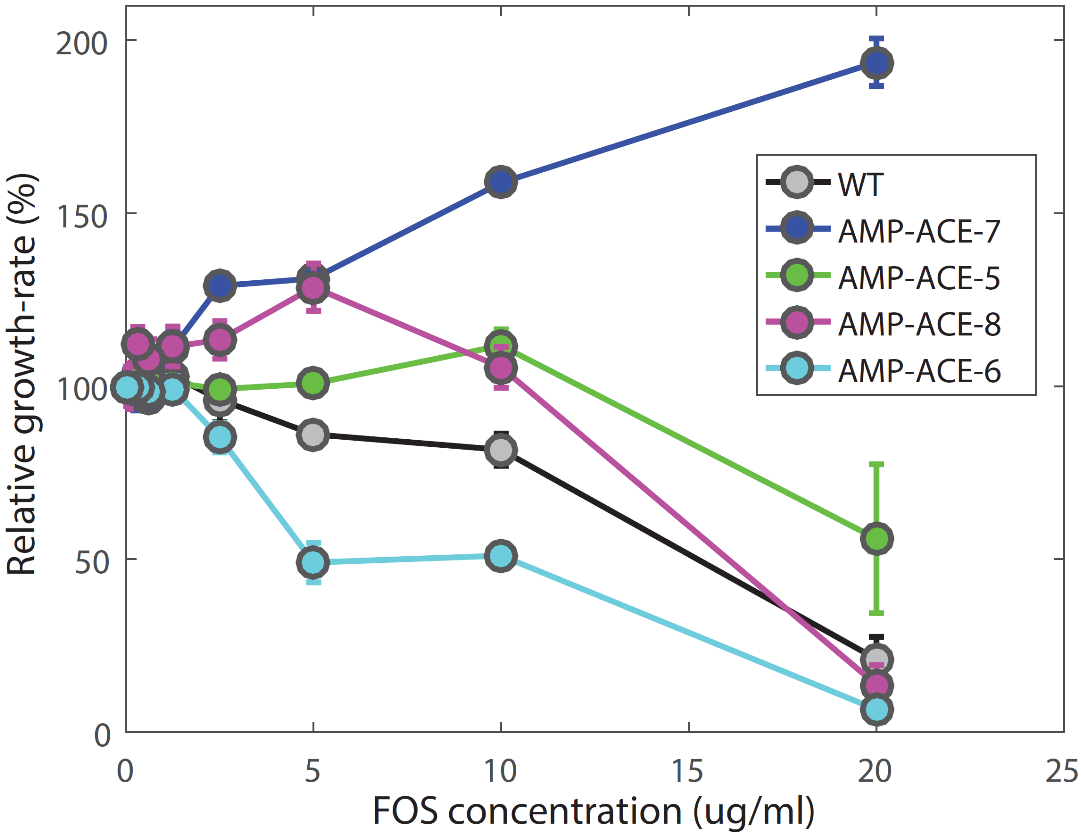

Figure S12: Sensitivity of ampicillin-acetate resistant populations to fosfomicyn (FOS). The relative growth rate inhibition of different FOS concentrations relative to antibiotic-free growth is reported for wild-type (wt) and the mutants evolved in the presence of ampicillin and acetate at the end point of the evolutionary experiment. Data are the mean ± S.D. of three biological replicates grown in acetate minimal medium. Overall, *E. coli* cells exhibited a higher tolerance to fosfomycin in acetate with respect to glucose minimal medium. Surprisingly, most of the evolved populations in acetate-ampicillin exhibited a growth advantage with relatively low fosfomycin concentrations, and one

population in particular (AMP-ACE-7) exhibited a strong benefit from the presence of fosfomycin. In this evolved population, growth-rate is 60% slower than wild-type *E. coli* in antibiotic-free acetate minimal medium, and it increases with higher fosfomycin concentrations.

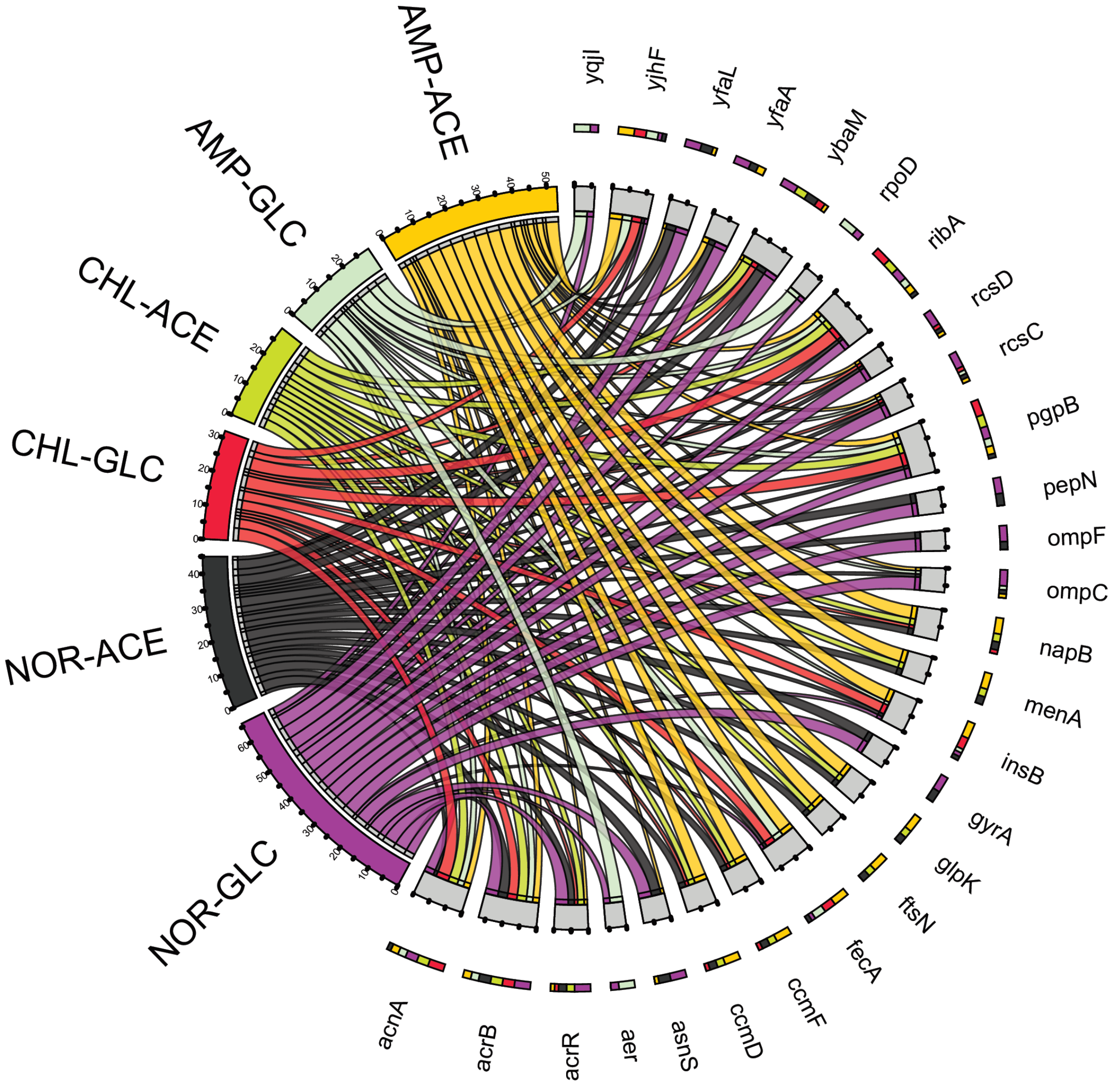

Figure S13: Common genetic changes identified by whole-genome sequencing. Mutations identified in at least two out of the four lineages evolved under the same selective pressure are retained. Only mutated genes in more than one condition are shown and linked to the respective selective antibiotic / carbon source.

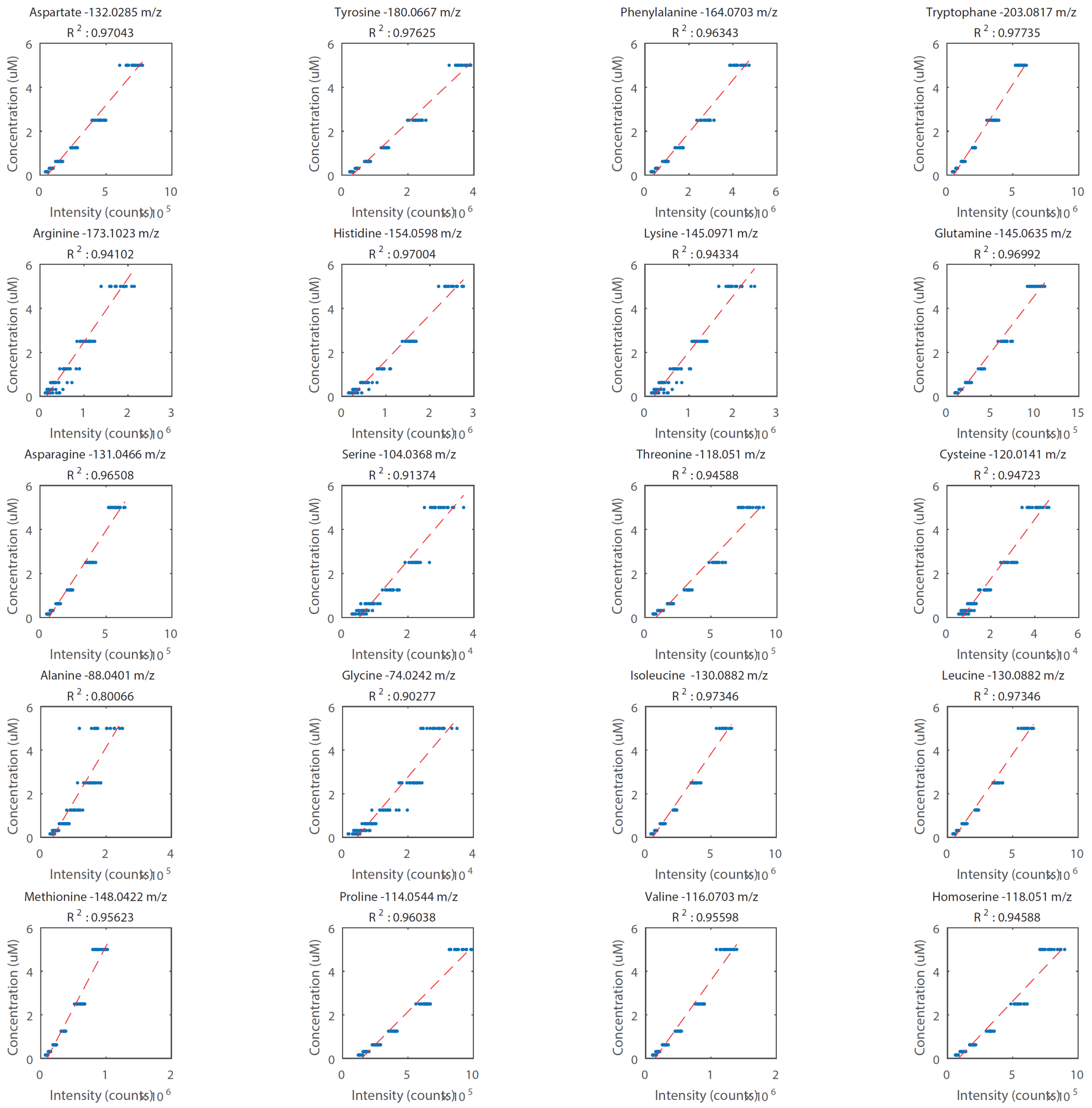
Aspartate -132.0285 m/z
R 2 : 0.97043
Tyrosine -180.0667 m/z
R 2 : 0.97625
Phenylalanine -164.0703 m/z
R 2 : 0.96343
Tryptophane -203.0817 m/z
R 2 : 0.97735
Arginine -173.1023 m/z
R 2 : 0.94102
Histidine -154.0598 m/z
R 2 : 0.97004
Lysine -145.0971 m/z
R 2 : 0.94334
Glutamine -145.0635 m/z
R 2 : 0.96992
Asparagine -131.0466 m/z
R 2 : 0.96508
Serine -104.0368 m/z
R 2 : 0.91374
Threonine -118.051 m/z
R 2 : 0.94588
Cysteine -120.0141 m/z
R 2 : 0.94723
Alanine -88.0401 m/z
R 2 : 0.80066
Glycine -74.0242 m/z
R 2 : 0.90277
Isoleucine -130.0882 m/z
R 2 : 0.97346
Leucine -130.0882 m/z
R 2 : 0.97346
Methionine -148.0422 m/z
R 2 : 0.95623
Proline -114.0544 m/z
R 2 : 0.96038
Valine -116.0703 m/z
R 2 : 0.95598
Homoserine -118.051 m/z
R 2 : 0.94588
Concentration (uM)
Intensity (counts)

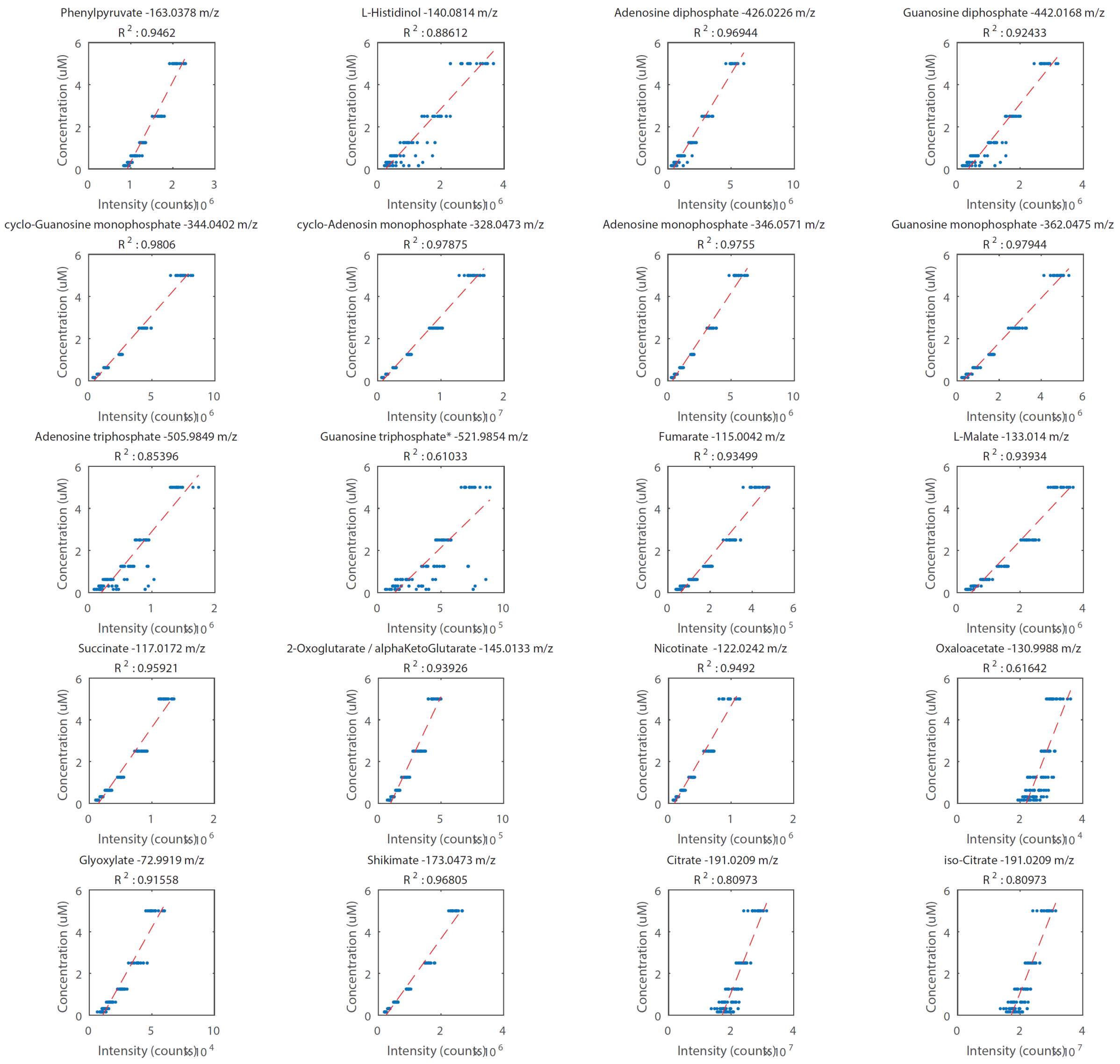
Phenylpyruvate -163.0378 m/z
R 2 : 0.9462
L-Histidinol -140.0814 m/z
R 2 : 0.88612
Adenosine diphosphate -426.0226 m/z
R 2 : 0.96944
Guanosine diphosphate -442.0168 m/z
R 2 : 0.92433
cyclo-Guanosine monophosphate -344.0402 m/z
R 2 : 0.9806
cyclo-Adenosin monophosphate -328.0473 m/z
R 2 : 0.97875
Adenosine monophosphate -346.0571 m/z
R 2 : 0.9755
Guanosine monophosphate -362.0475 m/z
R 2 : 0.97944
Adenosine triphosphate -505.9849 m/z
R 2 : 0.85396
Guanosine triphosphate* -521.9854 m/z
R 2 : 0.61033
Fumarate -115.0042 m/z
R 2 : 0.93499
L-Malate -133.014 m/z
R 2 : 0.93934
Succinate -117.0172 m/z
R 2 : 0.95921
2-Oxoglutarate / alphaKetoGlutarate -145.0133 m/z
R 2 : 0.93926
Nicotinate -122.0242 m/z
R 2 : 0.9492
Oxaloacetate -130.9988 m/z
R 2 : 0.61642
Glyoxylate -72.9919 m/z
R 2 : 0.91558
Shikimate -173.0473 m/z
R 2 : 0.96805
Citrate -191.0209 m/z
R 2 : 0.80973
iso-Citrate -191.0209 m/z
R 2 : 0.80973

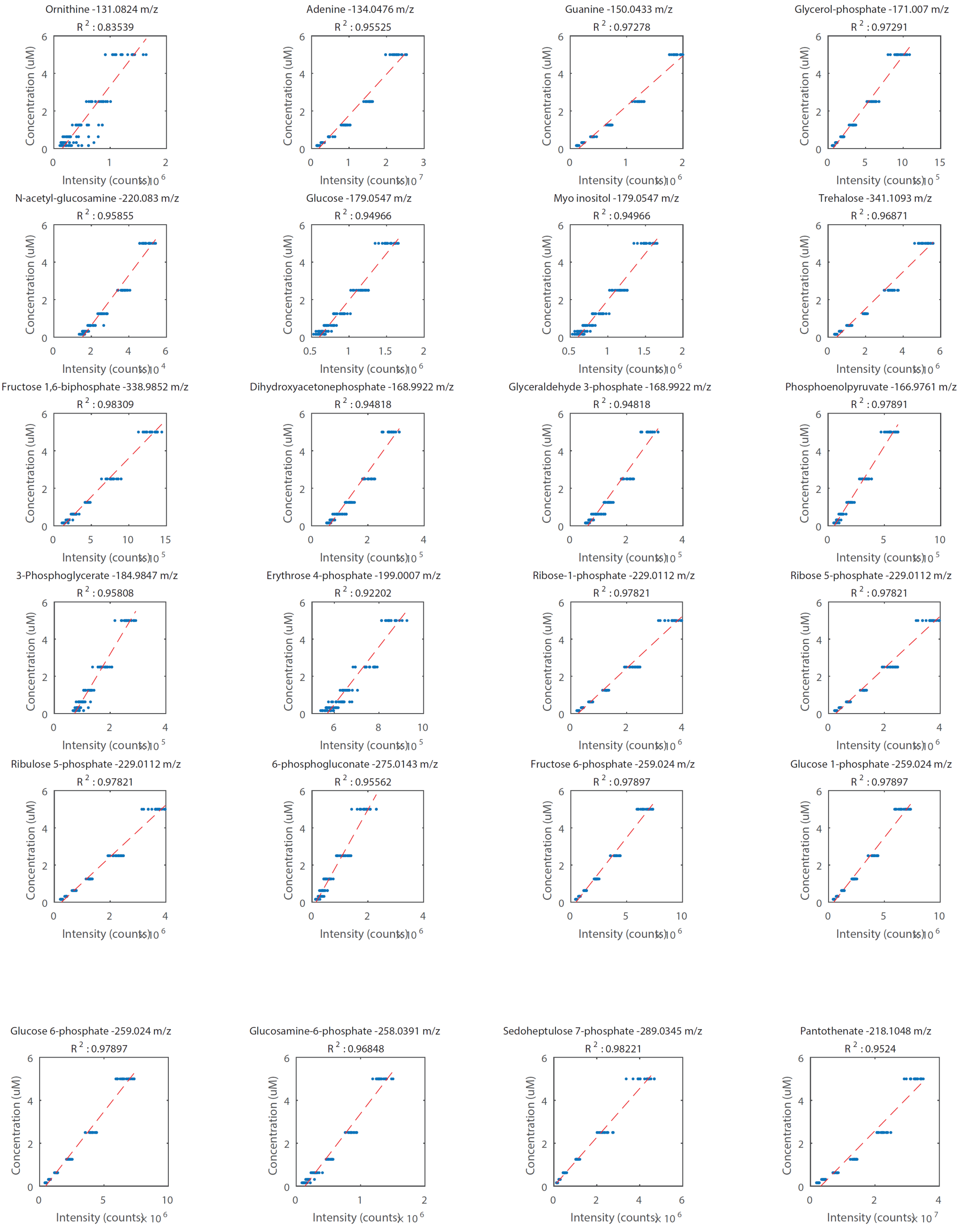

Figure S14: 64 different compounds purchased from Sigma were mixed in M9 minimal medium at an equimolar concentration of 100 µM. Samples were diluted to a concentration of 5, 2.5. 1.25, 0.625, 0.3125 and 0.1563 µM and measured intensities of peaks with an equivalent m/z are plotted against each

of the 64 compounds. Blu dots represent raw intensities from 6 spiking experiment (e.g. biological replicates) and 2 injections of the same sample (e.g. technical replicates). Linear regression analysis was performed (red dashed line) and adjusted R^2 are reported for each compound.

References

Baba T, Ara T, Hasegawa M, Takai Y, Okumura Y, Baba M, Datsenko KA, Tomita M, Wanner BL & Mori H (2006) Construction of Escherichia coli K-12 in-frame, single-gene knockout mutants: the Keio collection. *Mol. Syst. Biol.* **2:** 2006.0008

Basan M, Hui S, Okano H, Zhang Z, Shen Y, Williamson JR & Hwa T (2015) Overflow metabolism in Escherichia coli results from efficient proteome allocation. *Nature* **528:** 99–104

Bumann D & Valdivia RH (2007) Identification of host-induced pathogen genes by differential fluorescence induction reporter systems. *Nat. Protoc.* **2:** 770–777

König R, Chiang C, Tu BP, Yan SF, DeJesus PD, Romero A, Bergauer T, Orth A, Krueger U, Zhou Y & Chanda SK (2007) A probability-based approach for the analysis of large-scale RNAi screens. *Nat. Methods* **4:** 847–849

Li G-W, Burkhardt D, Gross C & Weissman JS (2014) Quantifying absolute protein synthesis rates reveals principles underlying allocation of cellular resources. *Cell* **157:** 624–635

Nichols RJ, Sen S, Choo YJ, Beltrao P, Zietek M, Chaba R, Lee S, Kazmierczak KM, Lee KJ, Wong A, Shales M, Lovett S, Winkler ME, Krogan NJ, Typas A & Gross CA (2011) Phenotypic landscape of a bacterial cell. *Cell* **144:** 143–156

Orth JD, Conrad TM, Na J, Lerman JA, Nam H, Feist AM & Palsson BØ (2011) A comprehensive genome-scale reconstruction of Escherichia coli metabolism--2011. *Mol. Syst. Biol.* **7:** 535

Storey JD (2002) A Direct Approach to False Discovery Rates. *J. R. Stat. Soc. Ser. B* **64:** 479–498
