# Supplementary material for: Metabolic constraints on the evolution of antibiotic resistance
Source: Mol Syst Biol. 2017 Mar 6;13(3):917. doi: 10.15252/msb.20167028 (PMC5371735; doi:10.15252/msb.20167028)
Supplement: Supplementary file 2 — Expanded View Figures PDF [file MSB-13-917-s002.pdf]

Expanded View Figures

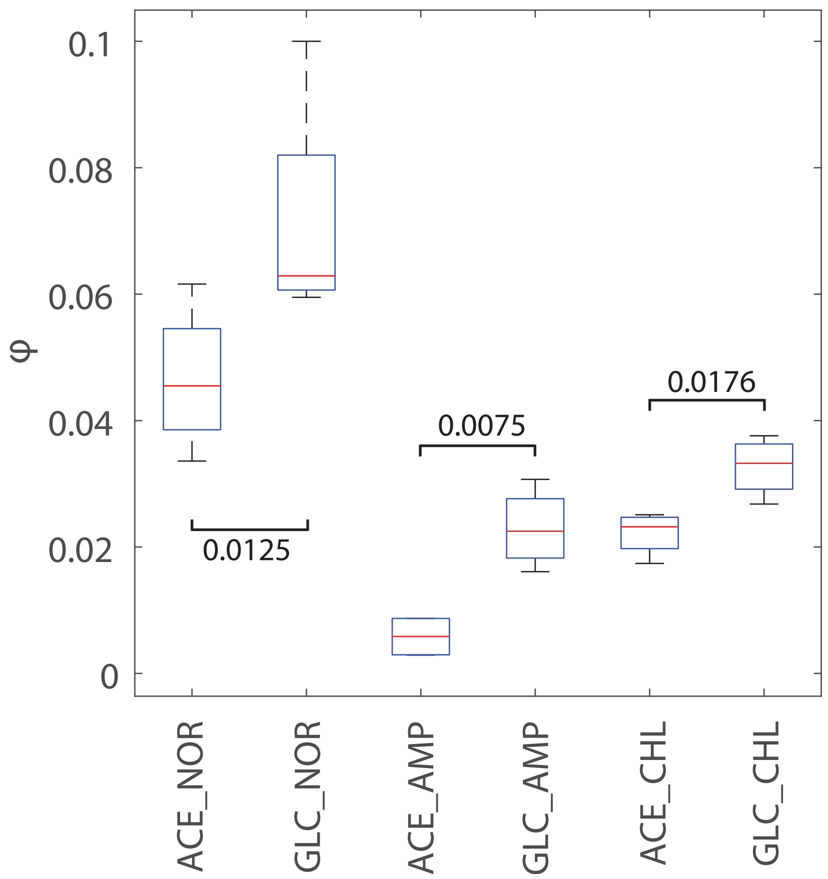

Figure EV1. Time constant estimate of speed of resistance evolution between glucose and acetate minimal media.

For each evolved lineage, the antibiotic concentration at which evolved populations were selected during serial passages (data reported in Fig 1C) are described by an exponential function: $D(g) = Ae^{\phi g}$, where D is the drug concentration at each passaging step, g is the number of generations, and ϕ and A are the fitted parameters. The estimates of ϕ for lineages evolved under the same selective pressure are grouped and their distribution plotted. For each group, the tops and bottoms of each box are the 25th and 75th percentiles, respectively, while the red line in the middle of each box is the samples median. The lines extending above and below each box are the whiskers. Whiskers extend from the ends of the boxes delimited by the interquartile to the largest and smallest observations. *P*-values of a *t*-test comparison between populations evolved under the same antibiotic pressure but in different media (i.e. glucose versus acetate) are reported.

© 2017 The Authors

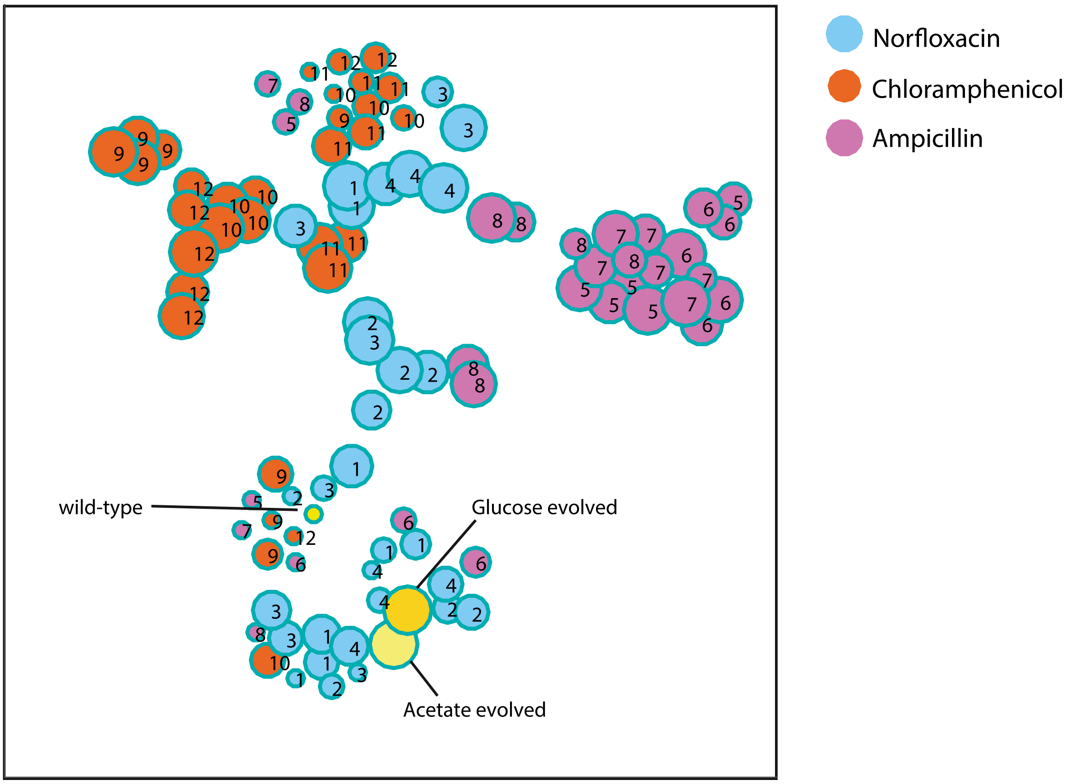

Figure EV2. Projection of high-dimensional metabolome profiles of glucose-evolved populations in a 2D-map.

t-Distributed Stochastic Neighbor Embedding (t-SNE; van der Maaten & Hinton, 2008) approach was here used to visualize the *Z*-score normalized metabolome profiles of evolved populations in glucose minimal medium. Similar to Fig 2, the 2D-map represents the square matrix of Spearman correlations (Fieller *et al*, 1957) between relative metabolite concentrations for each pair of evolved populations in a glucose minimal medium. Each dot represents one of the *Escherichia coli* populations selected for the 12 independent evolved lineages. Numbers indicate the respective lineage, and the size of the dots is proportional to the number of generations.

 © 2017 The Authors

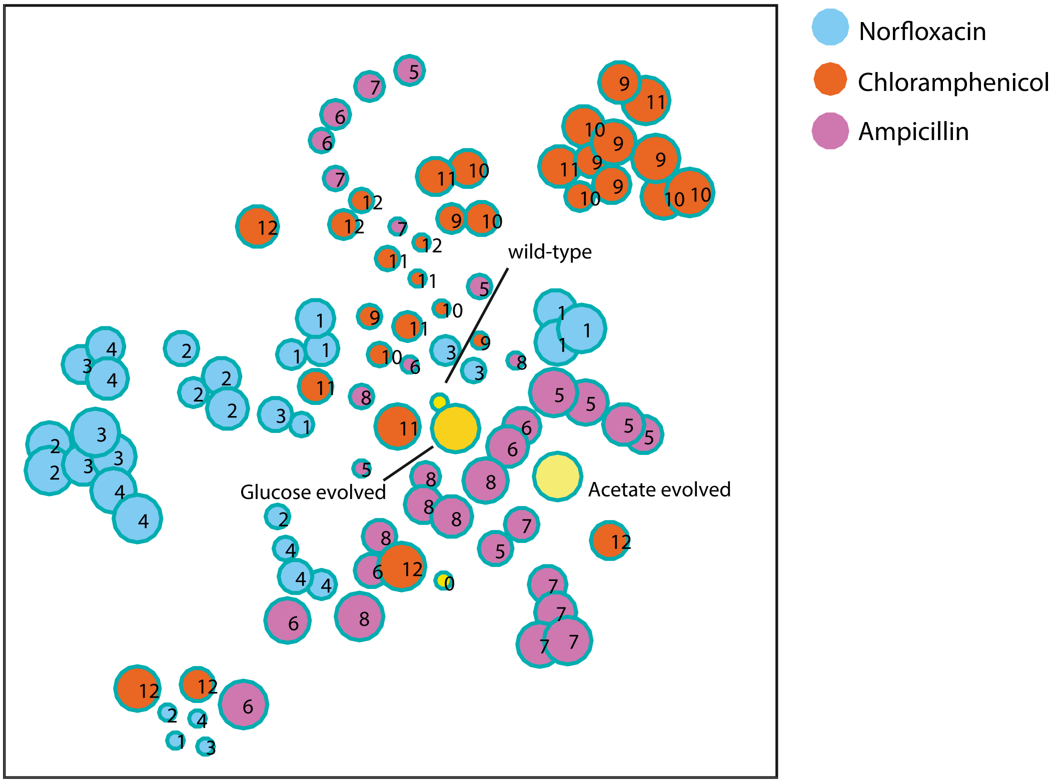

Figure EV3. Projection of high-dimensional metabolome profiles of acetate-evolved populations in a 2D-map.
t-Distributed Stochastic Neighbor Embedding (t-SNE; van der Maaten & Hinton, 2008) approach was here used to visualize the *Z*-score normalized metabolome profiles of evolved populations in acetate minimal medium. Similar to Fig 2, the 2D-map represents the square matrix of Spearman correlations (Fieller *et al*, 1957) between relative metabolite concentrations for each pair of evolved populations in a acetate minimal medium. Each dot represents one of the *Escherichia coli* populations selected for the 12 independent evolved lineages. Numbers indicate the respective lineage, and the size of the dots is proportional to the number of generations.

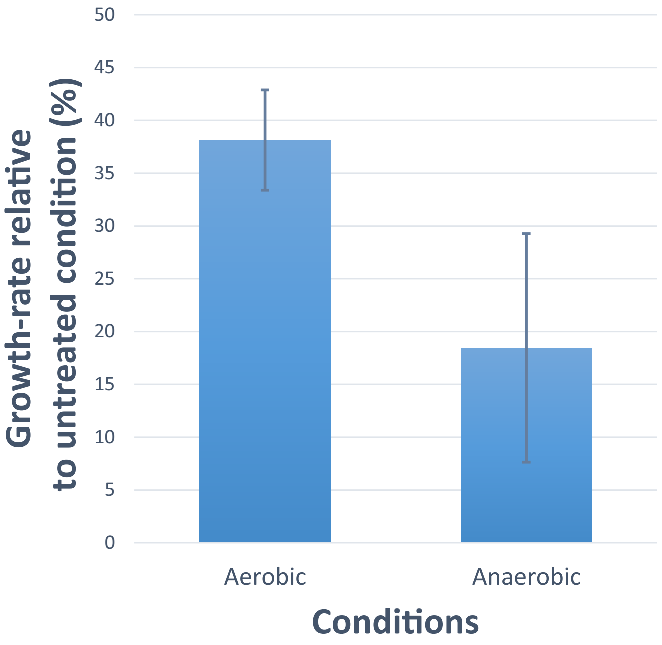

Figure EV4. Sensitivity to chloramphenicol in aerobic and oxygen-limited growth conditions.
Growth rate inhibition, estimated from optical density measurements (OD_{600}) in aerobic and oxygen-limited batch cultures, of wild-type *Escherichia coli* challenged with 4 μg/ml of chloramphenicol, relative to the respective normal conditions (i.e. anaerobic versus aerobic) without the antibiotic. Bars represent mean and standard deviation of three biological replicates. A comparison between the inhibitory activity of chloramphenicol between aerobic versus anaerobic conditions shows that chloramphenicol is less efficient upon oxygen limitation (*P*-value = 0.0447 from a *t*-test analysis).

© 2017 The Authors

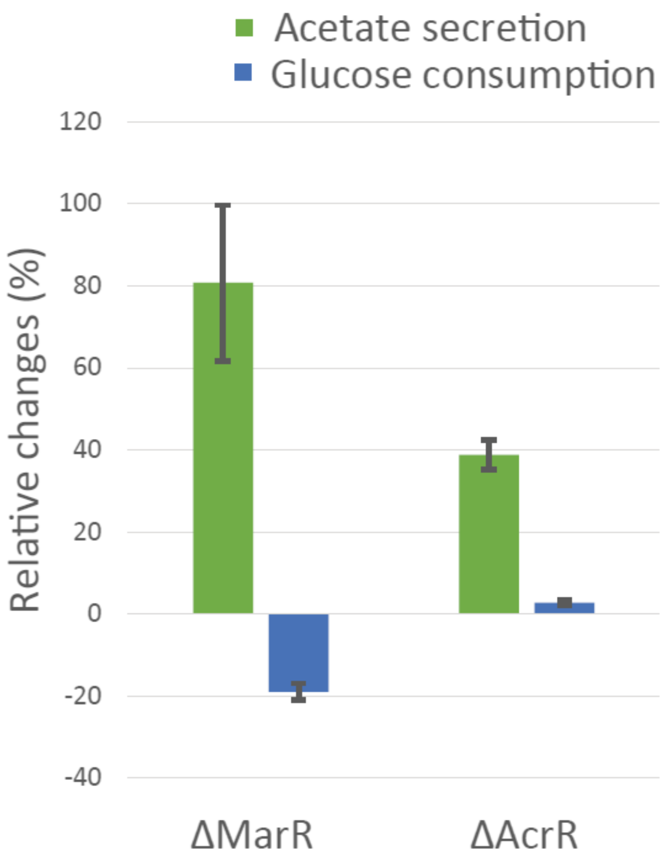

Figure EV5. Changes of acetate secretion and glucose consumption rates in *Escherichia coli* knockout strains Δ*marR* and Δ*acrR* with respect to wild-type *E. coli*.

Metabolic rates were measured using enzyme assay kit from Megazyme. Data are the mean ± SD of three biological replicates growing in a glucose minimal medium. Acetate secretion is significantly increased in Δ*marR* and Δ*acrR* strains, with a *P*-value of 0.0006 and 0.008, respectively.

 © 2017 The Authors
